# Novel ssDNA Ligand Against Ovarian Cancer Biomarker CA125 With Promising Diagnostic Potential

**DOI:** 10.3389/fchem.2020.00400

**Published:** 2020-05-15

**Authors:** Pranav Tripathi, Manisha Sachan, Seema Nara

**Affiliations:** Department of Biotechnology, Motilal Nehru National Institute of Technology Allahabad, Prayagraj, India

**Keywords:** CA125, aptainformatics, SELEX (systematic evolution of ligands by exponential enrichment), diagnostics, aptamer

## Abstract

The *in-vitro* diagnostic industry is always striving to explore specific and high-affinity recognition entities, sensitive probes, and newer technology or platforms to develop disease detection methods with lower production and time costs and with minimum interference or variability. Aptamers suffice as a reliable recognition element by addressing the issues mentioned earlier. Hence, this work focuses on screening high-affinity ssDNA ligands to capture an exemplary biomarker CA125 using membrane-SELEX technology coupled with aptainformatics (translational bioinformatics using aptamers). The ssDNA ligands or aptamers have been screened and characterized extensively through an array of assays to ensure a valuable diagnostic potential with K_D_ (dissociation constant) of 166 nM. The robustness of the selected aptamer ligand 2.26 and its complex with target CA125 is investigated in the presence of serum and extreme salt concentrations. Its diagnostic potential is convincingly demonstrated by running a competitive nucleic acid lateral flow assay at various sample concentrations. The ssDNA ligand reported in this manuscript holds immense potential in the detection and specific targeting of CA125 biomarker.

## Highlights

- High-affinity ssDNA aptamer to recognize CA125 biomarker.
- Most specific and robust ssDNA aptamer reported so far to capture native CA125.
- Highly stable in serum and at extreme salt concentrations.
- Its translational diagnostic potential is demonstrated in static as well as flow-through mode.

## Introduction

Since its inception, SELEX (Systematic Evolution of Ligands by Exponential enrichment) has assisted in identifying short sequences (Tuerk and Gold, 1990), which may be used as recognition elements in therapeutic or diagnostic applications. However, after obtaining a pool of sequences from the final cycle of SELEX, it is mandatory, as well as complex, to filter out better binding sequences with stable three-dimensional conformations to reduce the burden of testing each sequence through *in-vitro* methods. Further, it is observed that for a particular analyte, numbers of aptamer sequences selected through different SELEX approaches are globally reported. Hence, it is essential that case studies comparing such sequences binding to their targets are conducted and come out with the best recognition element. Aptainformatics plays a crucial role in meeting these requirements and may complement SELEX strategies by enriching or precisely narrowing down the pool of obtained sequences. This study also uses aptainformatics along with SELEX to screen a high-affinity aptamer sequence for CA125 (Lakhin et al., 2013).

CA125 is an FDA-approved biomarker used for non-invasive screening of ovarian cancer, which accounts for ~5% cancer deaths worldwide (ACS Ovarian Cancer News, 2018). To replace antibody-based CA125 ELISA, aptamers have been screened against native (Scoville et al., 2017) as well as recombinant CA125 (Lamberti et al., 2016; Gedi et al., 2018) by three different groups. CA125 is highly heterogeneous and is secreted as splicing variants ranging from 1,148 to 22,152 amino acids in length and from 200 to 5,000 kDa in size. Due to dissimilarities in repeat domains of these secreted variants, it is crucial to use native CA125 protein as the target rather than recombinant peptide for aptamer selection or assay design (Chen et al., 2017). Chen et al. used an aptamer that possessed the larger size and no focus was laid on the K_D_ of the aptamer, thus making it less efficient. Scoville et al. have used CA125 isolated from human ascites fluid but did not demonstrate the diagnostic potential of screened aptamers. Moreover, the method of computing the dissociation constant of reported aptamers by Scoville et al. also relied upon entities with two dissimilar units. Hence, this manuscript screens a high-affinity ssDNA aptamer for CA125 and demonstrates its translational potential as a capture reagent for CA125 detection through Dot ELASA (Enzyme-linked aptamer sorbent assay), DPV (Differential Pulse Voltammetry), and NALFA (Nucleic acid lateral flow assay). As a case study, aptamers screened in this manuscript are compared with previously reported DNA aptamers (Scoville et al., 2017) for their stability and binding with CA125 through an aptainformatics approach. As numerous aptamers are being developed for the same target but the binding sites are seldom studied, a comparison is least likely to be drawn for superiority and aptainformatics proves itself to be an excellent tool for such screening as well as comparison studies.

## Materials and Methods

All reagents and chemicals used were of analytical grade or HPLC grade. CA125 Native antigen from human ascites was purchased from MyBiosource, USA. Tetra chloroauric acid was purchased from Sigma-Aldrich, India. Monarch PCR & DNA clean up kit (5 μg) and Monarch DNA gel extraction kit was purchased from New England Biolabs, India. Hot start Taq Polymerase was procured from Thermo Fisher Scientific, and all membranes were purchased from MDI, India.

### Selection of Random DNA Library

Random N-30 ssDNA library and all primers were synthesized from Trilink Biotechnologies USA. The DNA template of the procured library was PO DNA [5′TAG GGA AGA GGA CAT ATG AT (N30)TTG ACT AGT ACA TGA CCA CTT GA 3′] where N indicates A, C, G, T wobble site. The sequence of primers complementary to the adaptors at 5′ and 3′ ends of the selected random library are as follows: forward selection primer PO DNA, 5′ TAG GGA AGA GAA GGA CAT ATG AT 3′ & reverse selection primer PO DNA, 5′ TCA AGT GGT CAT GTA CTA GTC AA 3′ or biotinylated Primers: 5′ Biotin forward selection primer PO DNA, 5′ (Biotin) TAG GGA AGA GAA GGA CAT ATG AT 3′ and 5′ Biotin reverse selection primer PO DNA, 5′ (Biotin) TCA AGT GGT CAT GTA CTA GTC AA3′.

### Membrane SELEX Methodology

Membrane-SELEX was accomplished using a 0.2 μm pore size nitrocellulose membrane and native CA125 antigen as the target. The blocking of membranes prior to SELEX was done using KOH and HMCKN buffer, as discussed by Frith et al. (2018). Twenty microliter of 400 pmol ssDNA library was denatured at 95°C for 10 min. To this, 400 μL of binding buffer and 20 μL of 830 U/mL CA125 was added and incubated at 25°C for 1 h. The binding buffer comprised of 10 mM PBS, 1 mM MgCl_2_, and 0.01% herring sperm DNA. This solution was passed through the blocked NC membrane, allowing the unbound or free sequences to pass through it. After three rounds of washing, CA125 bound sequences retained on the top of the NC membrane filter were treated with an elution buffer having 7 M urea as a denaturant to break their association with CA125. The eluent was collected, and oligonucleotides were extracted from it using Phenol: chloroform: isoamyl alcohol. Extracted sequences were PCR amplified. The PCR product of this first SELEX cycle was then subjected to the second round of membrane-SELEX after snap- chill, and the iterative process was repeated for six SELEX cycles. The last cycle was carried out by incubating the previous cycle product with only a nitrocellulose membrane for eliminating non-specific binding sequences. All the cycles of the SELEX process were carried out with the same designed protocol, except for variations in two parameters: incubation time with CA125 and its concentration. The PCR product of the last SELEX cycle was stored at −40°C for future use. PTZ57R/T vector was used for the insertion of obtained sequences from the final cycle of membrane SELEX. The ligated product was transformed into the DH5-α cells through heat shock immediately followed by chilled shock. Transformed colonies were selected through ampicillin/X-Gal/IPTG selection and reconfirmed through colony PCR. Automated DNA sequencing using the ABI 3500 DNA sequencing platform was performed for isolated plasmids from positive recombinant colonies. Post sequencing, membrane SELEX resulted in 140 clones.

### Aptainformatics Enrichment of SELEX Product

Aptainformatics enrichment of all putative aptamers was done *in-silico* using various computational tools. The secondary structure of aptamers was predicted through the M-fold web server. The modeling and validation of protein targets was accomplished using I-Tasser, mod loop, RAMPAGE, and PRO-SA. The RNA composer web server and Discovery studio visualizer were utilized to mutate and analyze their tertiary structure. The docking of the obtained 3D structure of selected ss-DNA and CA125 was performed with the help of the Patchdock and interactions were predicted using Ligplot. Molecular dynamics simulation of the CA125-aptamer complex was accomplished using the NAMD graphical interface module incorporated with visual molecular dynamics (VMD). The Automatic PSF builder tool of VMD was used to make the PSF file and by accessing PDB files. Further, NAMD generated the trajectory (.dcd) files by accessing psf and pdb files which were utilized to calculate the Root Mean Square Deviation (RMSD) of the complex. Rmsd.tcl source file was used to calculated RMSD from the Tk console, and rmsd.dat was obtained and accessed graphically.

### *In-vitro* Characterization of Serum Stability of Aptamers

One micrometer Apt2.26 was incubated both alone and in the presence of 50% v/v normal human female serum for 2 and 4 h subsequently at RT. The incubated samples were purified using the Phenol: Chloroform: Isoamyl alcohol based method and precipitated using 7.5 M ammonium acetate and absolute ethanol. The re-suspended purified aptamers were amplified using PCR and visualized on 2% agarose gel along with unamplified single-stranded templates.

### *In-vitro* Characterization of the Binding of Aptamers in the High Salt Incidence

One μM Apt2.26 was incubated in the presence of MiliQ water, 0.2 M NaHCO_3_, and 0.5 M NaCl as well as 100 mM NaCl and 5 mM MgCl_2_. 20 U/mL of CA125 was immobilized on a nitrocellulose membrane 4 mm in length and 4 mm in width, and blocked using 0.1% BSA. After washing, the incubated membranes were supplied with aptamers pre-incubated with salt. After 1 h of binding, the aptamers were eluted using 7.5 M Urea and 3 M sodium acetate followed by P:C:I purification and ethanol precipitation. Finally, the precipitated single-stranded aptamers were visualized on 2% agarose gel.

### Determination of Dissociation Constant (K_D_) for Aptamers

The binding affinity of aptamers against CA125 was elaborated by the equilibrium dissociation constant (K_D_), measured by estimation membrane-based assessment of bound ssDNA to CA125. The progressively diluted single strand aptamers (1 μM to 31 nM) in 3 μL of binding buffer were heated to 95°C for 5 min and immediately chilled at −20°C to form secondary structures. These aptamers were added to NC membrane pieces containing CA125 and further incubated for 2 h at RT. The bound aptamers were eluted using 7.5 M Urea and 3 M sodium acetate followed by P:C:I purification and ethanol precipitation. Aptamer concentration in each test tube was measured using a DeNovix DS-11 nanodrop machine. The K_D_ value was determined using a saturation binding curve upon the use of non-linear regression analysis in Prism software.

### *In-vitro* Binding Study Using Dot ELASA

To investigate the binding of CA125 with an aptamer, a particular concentration of CA125 was immobilized on nitrocellulose membrane. It was allowed to interact with two different concentrations of biotinylated Apt 2.26 (0.1 and 0.01 μM) for 60 min after blocking the membrane. The membrane was then washed and further incubated with Streptavidin-HRP, for 30 min. To this, DAB/H_2_O_2_ was added as a peroxidase substrate and results were visualized through the naked eye. Appropriate controls were kept in the assay.

### Synthesis, Characterization, and Conjugation of Gold Nanoparticles With CA125

Gold nanoparticles used as a label in this study were synthesized using trisodium citrate reduction as previously reported by us (Upadhyay and Nara, 2018). 0.8 M of boiling HAuCl_4_ salt (45 mL) and 1% trisodium citrate (5 mL) was added with vigorous stirring. The formed nanoparticles were characterized using UV-VIS spectroscopy, Agilent technologies, and measuring hydrodynamic diameter using dynamic light scattering system “Microtrac Nanotrac wave- II”. Conjugation of gold nanoparticles with CA125 was done by mixing 5 μL of 20 U/mL CA125 with gold nanoparticles (1 mL). After brief mixing, the solution was incubated with 0.01% Tween-20 and 0.1% BSA. Finally, after 2 h of incubation, the solution was centrifuged at 10,000 rpm and washed twice before resuspension in 10 mM phosphate buffer pH 7.4.

### *In-vitro* Binding Study Using NALFA

Ten micrometer nitrocellulose membrane laminate was utilized for the Nucleic acid lateral flow assay. One microliter of 10 μM aptamer was immobilized on NC laminate, and 8 μL of AuNPs- CA125 conjugate was applied on a reservoir matrix. The assay was run using PBS containing BSA and Tween-20. A competitive NALFA was also run using varying concentrations (0–200 U/mL) of CA125 in 0.1 M phosphate buffer as the test sample. Unlabeled CA125 in the test sample competed with AuNPs-labeled CA125. The color intensities of bands were recorded using the average RGB parameter of ImageJ software for quantification.

### *In-vitro* Binding Study Using Differential Pulse Voltammetry

The binding of CA125 with Apt 2.26 was also investigated using differential pulse voltammetry (DPV). Briefly, ITO chips (1.25 cm wide and 0.6 cm long) were washed with 10% NaOH for 10 min followed by washing with acetone. After drying, 20 μL of prepared GNPs were drop coated on the ITO surface and allowed to air dry at room temperature. To this GNP coated ITO chip, 0.25% glutaraldehyde was added and allowed to incubate for 20 min and washed with Milli-Q water. To this, 20 μL (20 U/ml) of CA125 was coated and incubated at room temperature for 60 min. After washing with Milli-Q water thrice, different concentrations of aptamer were drop coated on the ITO chips and washed thrice in Mili Q water after incubation for 60 min at room temperature. The conditions and parameters for DPV analysis were 100 mv/s scan rate, voltage window 0.0–1 V, and 5 mM ferri-ferrocyanide in 1 M KCl electrolyte.

## Results and Discussion

### Membrane-SELEX

**Figure 1:**
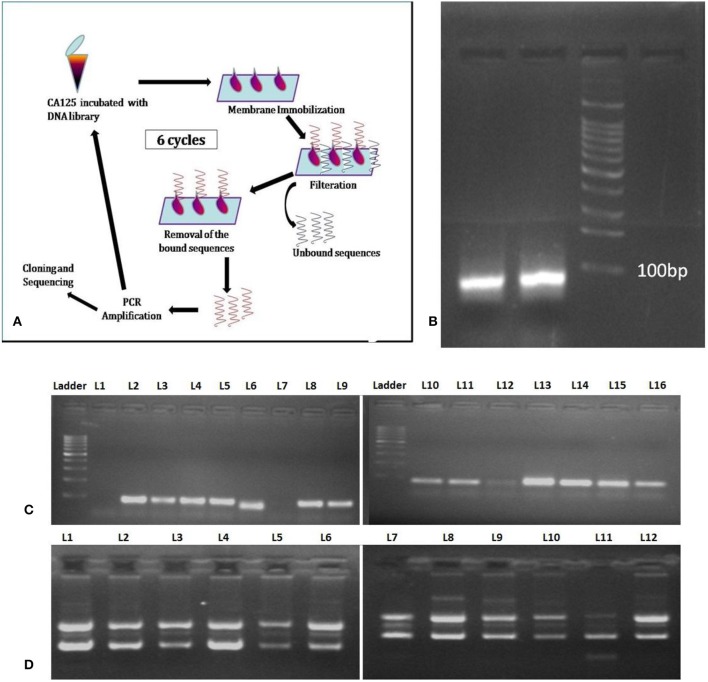
Membrane SELEX **(A)** General Schema. **(B)** PCR amplicon of last SELEX round, **(C)** colony PCR of selected colonies, and **(D)** plasmids isolated from selected colonies and used for sequencing. The original gel images have been provided in Figures S1–S3.

Membrane-SELEX (Figure 1A) was accomplished using 0.2 μm pore size nitrocellulose membrane and native CA125 antigen as the target. The PCR amplified product of the first SELEX cycle was then subjected to the subsequent cycles of membrane-SELEX. Five such iterative cycles were performed with CA125, and the last cycle was subtractive to mitigate the non-specifically binding sequences (Table 1). CA125 concentration and/ or time of incubation of ssDNA with CA125 varied across different iterative rounds. The final round of ssDNA was PCR amplified (Figure 1B), cloned in a PTZ57R/T vector, and transformed into *E. coli* DH5-α through heat shock (Figure 1C). Automated DNA sequencing using ABI 3500 DNA sequencing platform was performed for plasmids isolated (Figure 1D) from 140 positive recombinant colonies. The obtained sequences were investigated for the presence of the complete library sequence. The sequences were collapsed on the basis of unique reads and number of times a sequence was represented. All 37 sequences appeared as unique reads and hence these 37 sequences were then analyzed in detail through aptainformatics. The original gel images have been provided in Figures S1–S3.

**Table 1:** Conditions used during different rounds of SELEX.

**Round**	**Positive/negative**	**Target**	**[Target]**	**DNA source**	**[DNA]**
1	Positive	CA125	16.6 U	Library	400 μM
2	Positive	CA125	16.6 U	Round 1 product	100 μM
3	Positive	CA125	8.3 U	Round 2 product	50 μM
4	Positive	CA125	8.3 U	Round 3 product	10 μM
5	Positive	CA125	4.15 U	Round 4 product	500 nM
6	Negative	Membrane	0 U	Round 5 product	50 nM

### Aptainformatics Enrichment of SELEX Product

**Figure 2:**
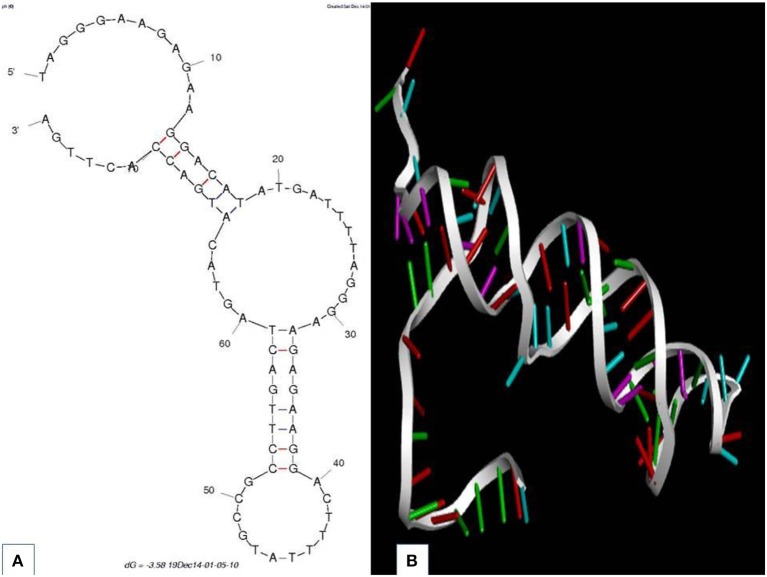
Aptamer 2.26 **(A)** predicted secondary structure with the corresponding Gibbs free energy. **(B)** Tertiary structure.

Aptainformatics enrichment of all putative aptamers was done *in-silico* using various computational tools. The secondary structure of aptamers was predicted through the mfold web server at 25°C (Zuker, 2003). The secondary structure of all the obtained sequences was predicted using the mfold server at ionic conditions of 1 M Na^+^ with an upper bound limit of 50 on the number of foldings at 5% suboptimality. None of these sequences had any typical loop in their secondary structure. An exemplary secondary structure with the most favorable ΔG is depicted in Figure 2A. The modeling and validation of the protein target were accomplished using I-Tasser, modloop, RAMPAGE, and PRO-SA. The RNA composer web server and Discovery studio visualizer were utilized to mutate and analyze their tertiary structure. The 3-D structures of the sequences mentioned above were modeled using RNA Composer (Antczak et al., 2016) and were mutated to DNA using Discovery studio visualize (Figure 2B) (Dassault Systèmes, 2016)

**Figure 3:**
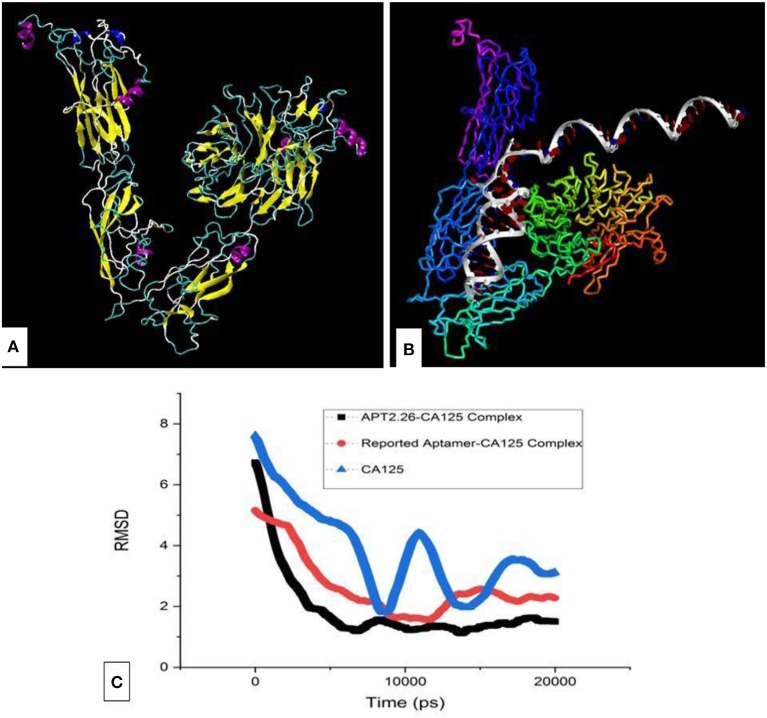
**(A)** The tertiary structure of the antigenic domain of CA125 and its **(B)** complex with aptamer 2.26. **(C)** Molecular dynamics simulation of CA125 alone as well as in complex with Apt 2.26 and reported aptamer, respectively.

CA125 native antigen is a large protein with ~22,000 amino acids and thus cannot be modeled entirely. Hence, it's one of the crucial antigenic domains (13,361–14,347 residues) that was modeled Ab-initio using I-Tasser (Yang et al., 2015). We referred to a recombinant primary sequence of CA125 protein which is commercially available [R&D systems a biotechne brand, Catalog no. 5609-MU-050] and whose binding ability has been validated with functional ELISA. Since this stretch of amino acid is present in the native protein as well, it seems justified to use this stretch for creating an *in silico* model and use it in the docking studies. The modeled structure (Figure 3A) was validated using Ramachandran plot analysis through RAMPAGE (Lovell et al., 2002) and PRO-SA webserver (Wiederstein and Sippl, 2007) and resulted in a *Z*-score of −5.62. The docking of obtained 3D structures of selected ss-DNA and CA125 was performed with the help of the Patchdock and interactions were predicted using Ligplot. Binding analysis of CA125 with the tertiary structure of all 37 aptamers was performed through multiple runs on the patch dock server (Figure 3B) (Schneidman et al., 2005). The algorithm of patch dock depicts binding scores that are proportional to binding affinities. A higher binding score is indicative of better binding between target-ligand and hence, is used as a criterion to screen better binders (Islam et al., 2019).

Hence, based on binding scores, four aptamers (Apt 2.13, Apt 2.43, Apt 2.26, and Apt 1.10) were screened to analyze their probable cross-reaction with Bovine Serum Albumin (BSA), Human Serum Albumin (HSA), and Immunoglobulin (IgG) on the same platform. These bindings, as well as cross-reactivity studies, were also performed with a previously reported CA125 DNA aptamer (Scoville et al., 2017). The binding score, as depicted in Table 2, indicates that Aptamer Apt2.26 has a maximum binding affinity for CA125 and low affinity for BSA, has, and IgG. Thus, Apt2.26 showed specific and robust binding toward CA125 (binding score of 21,286) in comparison to other sequences screened by us as well as with that of the previously reported aptamer. Therefore, the residues involved in the apt2.26-CA125 interaction were analyzed with LIGPLOT (Wallace et al., 1996) and are listed in Table 3. It was observed that 14 amino acids of CA125 were engaged in establishing hydrophobic interactions while five amino acids established covalent interactions with Apt 2.26; such interactions provide a firm base to the complex toward stability. After an affirmation of good hydrophobic and covalent interactions, the stability was re-checked using molecular dynamics simulation to ensure a real-world situation. Molecular dynamics simulation of CA125-aptamer complex was accomplished using the NAMD graphical interface module incorporated in visual molecular dynamics (VMD). The simulation used NAMD (Phillips et al., 2005) set at a default parameter for 20 ns and the RMSD data analysis showed that RMSD for APT2.26-CA125 complex was 1.499 Å, whereas the previously reported aptamer-CA125 complex showed an RMSD of 2.28 Å. However, CA125 itself showed a subtle pattern resulting in an RMSD of 3.22 Å with further chances of fold change, as shown in Figure 3C. So it can be inferred that the stability of the apt 2.26-CA125 complex is higher than that of the aptamer reported in the literature and hence the Apt 2.26 screened in this study could be a better recognition element for CA125. Table S1 of supplementary information tabulates the occurrence wise detailed sequences of highest frequency aptamers.

**Table 2:** Binding scores of aptamer candidates with CA125, BSA, HSA, and IgG.

**Sl no**.	**Aptamer**	**CA125 binding score**	**BSA binding score**	**HSA binding score**	**IgG binding score**
1	Apt2.43	21,087	18,941	21,029	15,718
2	Apt2.26	21,286	15,430	13,279	14,749
3	Apt1.10	19,083	16,530	18,199	14,532
4	Apt2.13	19,790	17,468	21,621	16,919
5	Reported aptamer	19,386	13,242	17,378	16,339

**Table 3:** Binding pockets and various interactions in Apt 2.26-CA125 complex.

**Amino acid residues forming hydrophobic interactions in Apt 2.26-CA125 complex**	
Val901, Met898, Ser900, Pro649, Lys651, Leu677, Thr723, Glu362, Lys416, Phe237, Leu364, Tyr365, Gly586, and Leu588	
**Covalently interacting residues of Apt 2.26-CA125 complex**	
**Residue**	**Interacting base**
ASP899	A37 and G38
ILE650	G40
ARG361	G42
PRO335	G44
SER587	G53

### Estimation of Serum Stability and Binding of Aptamers in the High Salt Incidence

**Figure 4:**
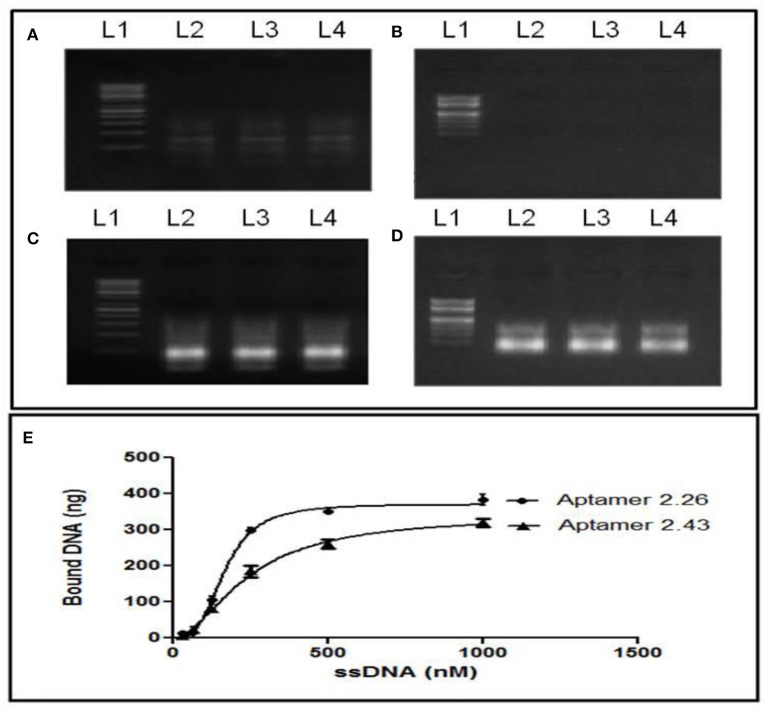
Serum stability profiling: **(A)** PCR amplified DNA and **(B)** its single-stranded form before amplification, after treatment with 50%v/v normal human female serum. Effect of different salt concentrations on aptamer-CA125 binding: **(C)** negative controls and **(D)** treated aptamer (lane 1: ladder, lane 2: 0.2 M NaHCO_3_ with 0.5 M NaCl, lane 3: 100 mM NaCl and 5 mM MgCl_2_, lane 4: milli-Q water as positive control; **(E)** Binding—saturation curve for determination of K_D_. The original gel images have been provided in Figures S4, S5.

The stability of aptamers in serum is a significant parameter if its applications are intended for serum-based diagnostics or therapeutics because any nucleases in the serum may degrade the aptamer or affect their stability. One micrometer Apt2.26 was incubated alone and in the presence of 50% v/v normal human female serum for 2 and 4 h at RT. Then, purified aptamer was visualized on 2% agarose gel before and after PCR amplification. Incubation of apt 2.26 with 50% v/v normal human female serum did not affect its stability with varying time of incubation as compared with the control (Figure 4B, Lane 2 = 2 h, Lane 3 = 4 h, Lane 4 = control). A shift in the position of bands in PCR-amplified (Figure 4A) and non-amplified sequences (Figure 4B) are attributed to the presence of dsDNA and ssDNA, respectively. This investigation establishes the future use of this aptamer in serum-based diagnostics. Target-aptamer binding is greatly influenced by changing salt types or concentrations. Hence, the binding of Apt2.26 with CA125 is also assessed in the presence of various salts. Briefly, 1 μM Apt2.26 was prepared in (a) MiliQ water, (b) 0.2 M NaHCO_3_ with 0.5 M NaCl, and (c) 100 mM NaCl with 5 mM MgCl_2_. Figure 4D depicts single strand aptamer 2.26 exposed to 0.2 M NaHCO_3_ with 0.5 M NaCl (lane 2), 100 mM NaCl with 5 mM MgCl_2_ (lane 3), and milli-Q water as positive control (lane 4). Figure 4C depicts corresponding negative controls for each salt group. MgCl_2_ is kept to facilitate the aptamer target binding. However, these results indicate that aptamer 2.26 is highly stable even at very high salt concentrations, i.e., 0.5 M NaCl in the absence of MgCl_2_. The binding of the aptamer is specific to CA125 as evident for the absence of bands in all negative controls. The original gel images have been provided in Figures S4, S5.

### Determination of Dissociation Constant (K_D_)

The binding affinity of aptamers against CA125 was determined by measuring its equilibrium dissociation constant (K_D_). CA125 (20 U/mL) immobilized NC membranes were dipped in various aptamer concentrations (1 μM−31 nM) after snap chilling. The bound aptamers were eluted using 7.5 M Urea and 3 M sodium acetate followed by ethanol precipitation. Concentrations of bound and unbound aptamers were measured using DeNovix DS-11 nanodrop machine. The K_D_ value was determined using a saturation binding curve upon the use of “one site-specific binding” non-linear regression analysis in Prism software (Joeng et al., 2009). The determination of the K_D_ analysis curve resulted in a robust sum of squares of 1.041. The concentration of CA125-bound aptamer was then plotted against the total aptamers used (Figure 4E). K_D_ was determined using equation 1 derived from the plot and was calculated to be 166 and 239.3 nM for Apt 2.26 and Apt 2.43, respectively.

$$\begin{array}{r} y=Bmax^{*}freessDNA/K_{\text{D}}+freessDNA \end{array}$$

### Diagnostic Potential of the Screened Aptamer

**Figure 5:**
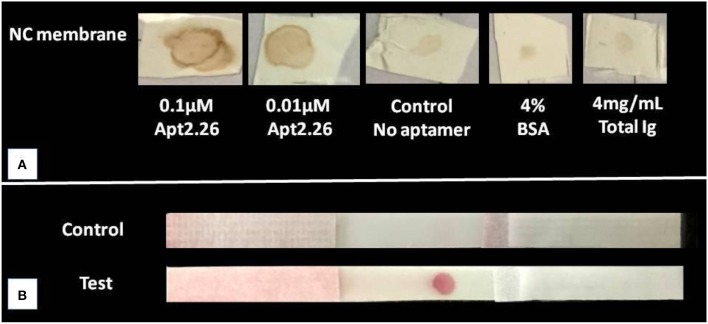
Binding study of CA125 with aptamer 2.26 using **(A)** Dot ELASA where CA125 was immobilized on nitrocellulose membrane, blocked, and then incubated with biotinylated apt2.26 followed by incubation with streptavidin-HRP. DAB/H_2_O_2_ was used as a peroxidase substrate resulting in a brown colored product. **(B)** NALFA (*N* = 4 for both assays) where 10 μM aptamer was immobilized on NC laminate, and AuNPs- CA125 conjugate was applied on a reservoir matrix. The assay was run using PBS containing BSA and Tween-20.

Next, we wanted to investigate whether aptamer 2.26 possesses significant diagnostic potential which could be translated further for designing a point of care diagnostic method for CA125 detection. This study was carried out in static as well as flow-through conditions using dot ELASA and NALFA to ensure that the aptamer can recognize its target in 3–5 min during flow conditions. For the dot ELASA, CA125 was immobilized on a nitrocellulose membrane and appropriately blocked for non- specific binding sites. It was allowed to incubate with biotinylated apt2.26, washed, and subsequently incubated with streptavidin-HRP. After washing, DAB/H_2_O_2_ was used as a peroxidase substrate resulting in a brown colored product (Qian and Huang, 2010). An intense brown color spot confirms significant binding between Apt 2.26 and CA125 in both 0.1 and 0.01 μM concentrations of apt2.26 tested as compared with negative control, 4% BSA, and 4 mg/mL total human immunoglobulins (Figure 5A).

APT2.26-CA125 binding was further validated in a flow-through condition by conducting NALFA. Gold nanoparticles (AuNP) of ~16 nm diameter were synthesized using the trisodium citrate reduction method, and they were used to label CA125 protein (Singh et al., 2015) The characterization results of gold nanoparticles using UV-Vis spectroscopy, TEM, and dynamic light scattering have been depicted in Figure S6 (supplementary information). To conduct NALFA, Apt2.26 was immobilized on a nitrocellulose membrane laminate of 10 μm pore size at the test zone. CA125-AuNP was coated on a reservoir matrix, and all components of the NALFA strip were assembled. The NALFA strip was run using 140 μL of running buffer. A prominent red color appeared in the test zone, confirming binding between Apt 2.26, and labeled CA125 (Figure 5B). The results were obtained within 3–5 min, indicating a high-affinity interaction between the two entities and ensuring that aptamer does not wipe out during the flow from the membrane. Absence of any red color in the negative strips with no immobilized aptamer also confirms a specific interaction between the screened aptamer and target CA125.

**Figure 6:**
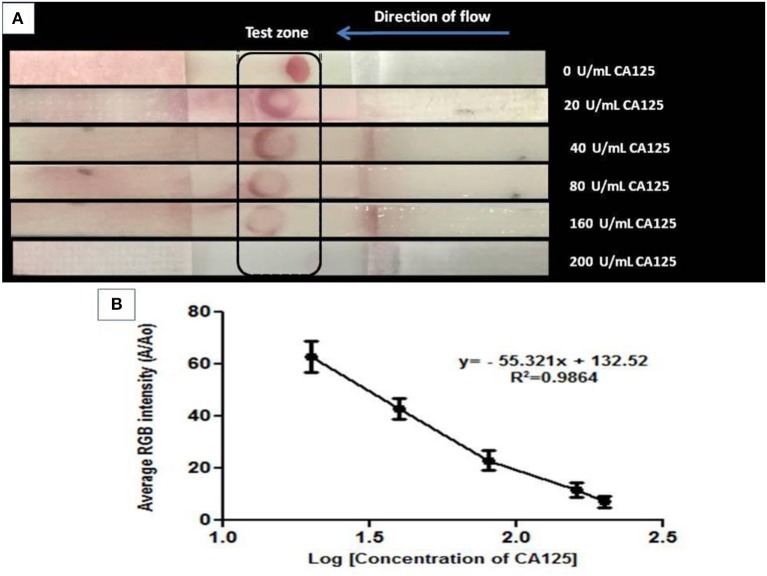
**(A)** Visual detection of CA125 at its gradually increasing concentrations in competitive format through NALFA where 10 μM aptamer was immobilized on NC laminate, and AuNPs- CA125 conjugate was applied on a reservoir matrix. The assay was run using varying concentrations (0–200 U/mL) of CA125 in 0.1 M phosphate buffer as the test sample. **(B)** Quantification of NALFA using ImageJ software by calculating average RGB intensities.

A competitive NALFA was also run using varying concentrations (0–200 U/mL) of CA125 in 0.1 M phosphate buffer as the test sample. Unlabeled CA125 in the test sample competed with AuNPs-labeled CA125. A gradual decrease in color intensity at the test zone (Figure 6A) with increasing CA125 concentration in the test sample is seen by visual detection and confirms gradually increasing competition between the unlabeled and labeled CA125. The decrease in RGB intensity can be visualized in Figure 6B. Three different concentrations of BSA (5, 10, and 15 mg/mL) were also used in the NALFA test and results were plotted using Prism 5. The selectivity test shows no cross-reaction with BSA. Data has been provided in Figure S7 of supplementary information. This assay could be presented as a proof of concept for rapid detection and quantification of CA125 using aptamer 2.26 and further establishes the strong translational potential of this aptamer in CA125 detection.

**Figure 7:**
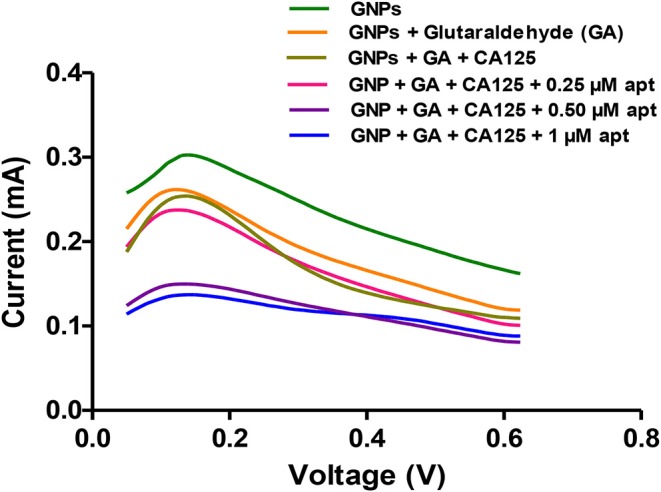
Apt 2.26-CA125 binding using differential pulse voltammetry.

Further, the binding of selected Apt 2.26 was investigated at different concentrations (0–1 μM) with 20 U/mL of CA125 using differential pulse voltammetry. The current values were plotted against the voltage window of 0–1 V. Figure 7 depicts a decrease in the values of current with decreasing concentrations of aptamer. A sharp decline in the current at 0.50 μM aptamer concentration confirms its binding with CA125.

## Conclusions

This work reports an ssDNA aptamer as a ligand to recognize and capture CA125 biomarker with high affinity and demonstrates its diagnostic potential in both static and flow-through conditions. Aptamer 2.26 (5′TAGGGAAGAGAAGGACATATGATTTTAGGGAAGAGAAGGACTTTTATGCCGCCTTGACTAGTACATGACCACTTGA3′) is demonstrated here as a better candidate than previously reported DNA aptamer for specifically capturing native CA125. The binding study of Apt 2.26 with CA125 as investigated using three *in vitro* methods including DOT ELASA, NALFA, and DPV to demonstrate the diagnostic potential of the screened aptamer. Its stability in human serum and high salt concentrations further proves its robust nature for use with complex sample matrices. The emerging trends in the diagnostic sector are toward the point of care technology (POCT), which is expected to increase by 140.4%, and LFA technology specifically by 45% by 2023 (Lateral Flow Assay Market, 2018). Hence, our team is working further to explore the applications of aptamer 2.26 in point of care diagnostics as well as therapeutics to contribute toward affordable healthcare in remote settings in developing nations.

## Data Availability Statement

The raw data supporting the conclusions of this article will be made available by the authors, without undue reservation.

## Ethics Statement

The current study used clinical human serum samples after prior clearance from Institute Ethical committee vide reference number IEC/2019-20/03.

## Author Contributions

PT is a research scholar working on this study as a part of his doctoral thesis. He has executed all experiments, analyzed and compiled the findings, and written this manuscript. MS has helped to plan molecular biology related experiments and troubleshoot (if any). SN has supervised the whole study from planning to execution and result analysis.

## Conflict of Interest

The authors declare that the research was conducted in the absence of any commercial or financial relationships that could be construed as a potential conflict of interest.
