# Supplementary material for: Novel ssDNA Ligand Against Ovarian Cancer Biomarker CA125 With Promising Diagnostic Potential
Source: Front Chem. 2020 May 15;8:400. doi: 10.3389/fchem.2020.00400 (PMC7242751; doi:10.3389/fchem.2020.00400)
Supplement: Supplementary file 1 [file Data_Sheet_1.docx]

**
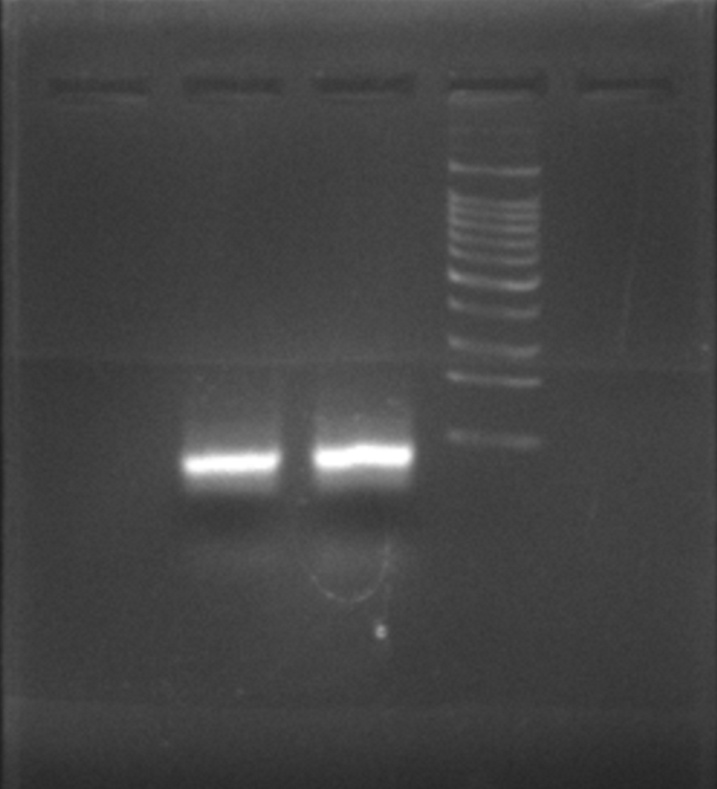
**

Figure S1. Original gel image (Figure 1B): PCR amplicon of last SELEX round


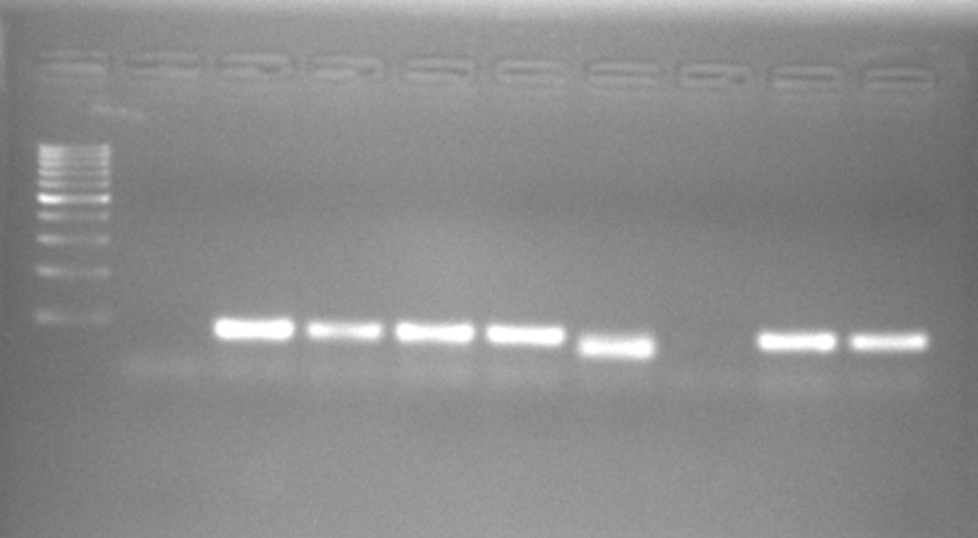

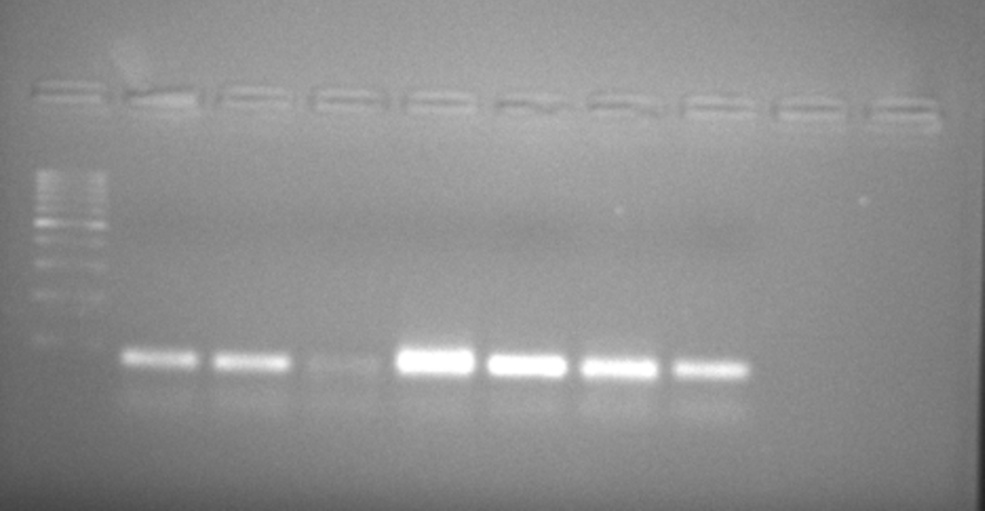


B

A

Figure S2. A & B) Original gel image (Figure 1C): colony PCR of selected colonies


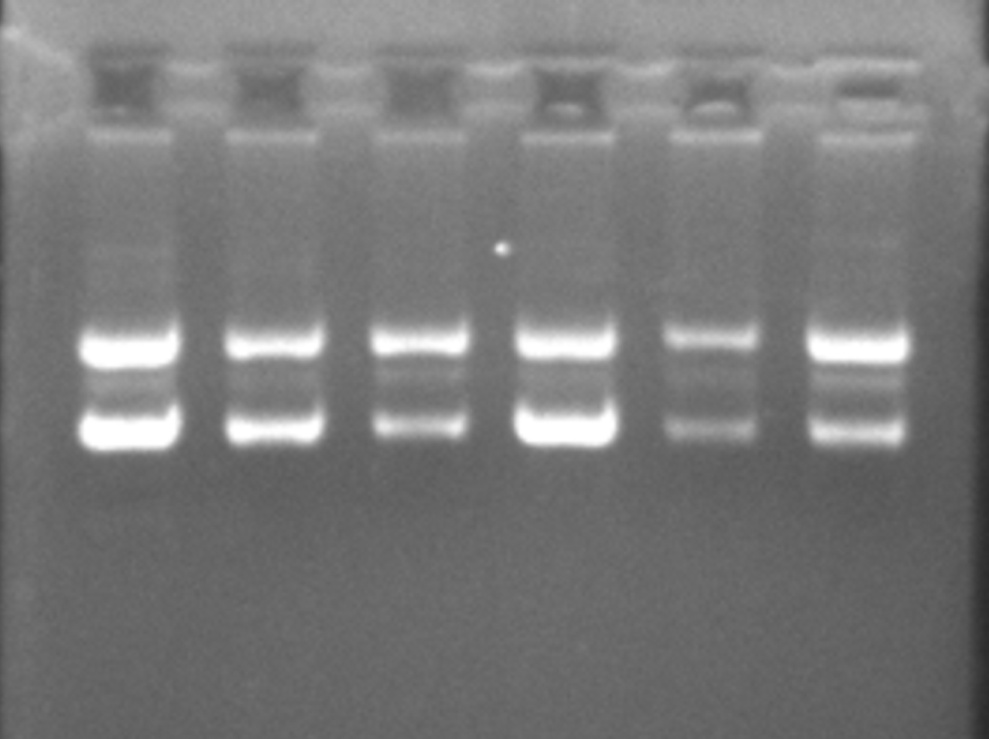

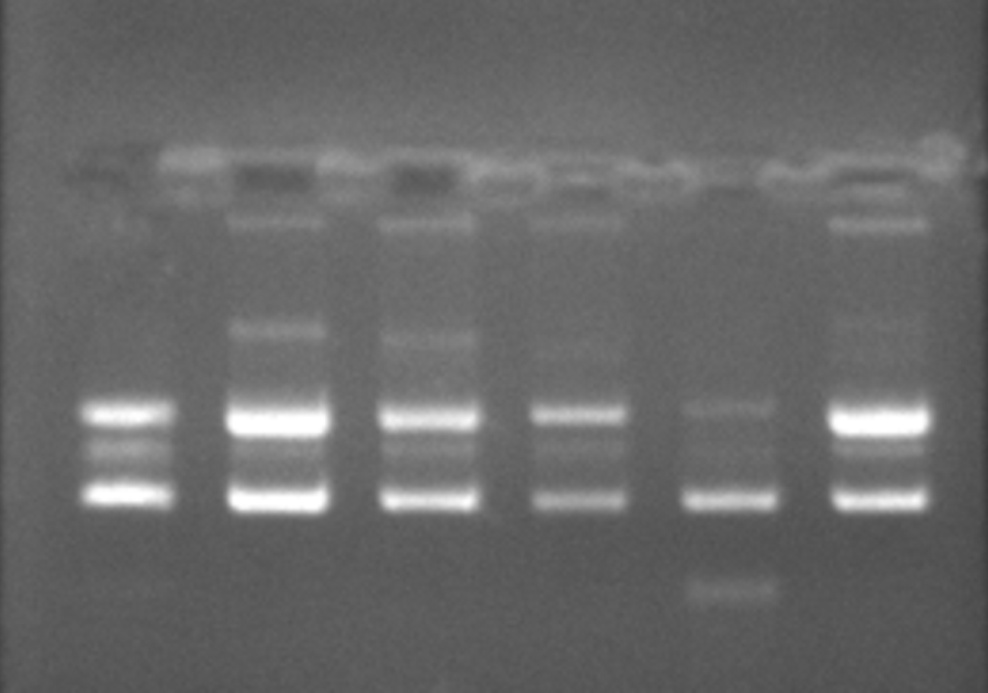


B

A

Figure S3. A & B) Original gel image (Figure 1D): plasmids isolated from selected colonies and used for sequencing.


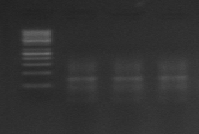

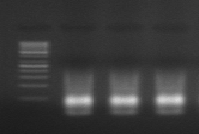


B

A

Figure S4. Original gel image (Figure 4 A & B): Serum stability profiling: (A)PCR amplified DNA and (B) its single stranded form before amplification, after treatment with 50%v/v normal human female serum.


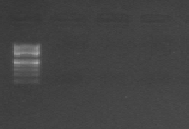

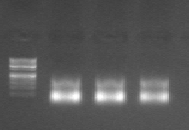


B

A

Figure S5. Original gel image Original gel image (Figure 4 C & D): the effect of different salt concentrations on aptamer-CA125 binding: (A) negative controls and (B) treated aptamer (lane 1: ladder, lane 2: 0.2M NaHCO_3_ with 0.5M NaCl, lane 3: 100mM NaCl and 5mM MgCl_2_*_,_*lane 4: milli-Q water as positive control;

Figure S6. Characterization of gold nanoparticles using (A) UV-Vis spectroscopy, (B) dynamic light scattering and (C) TEM


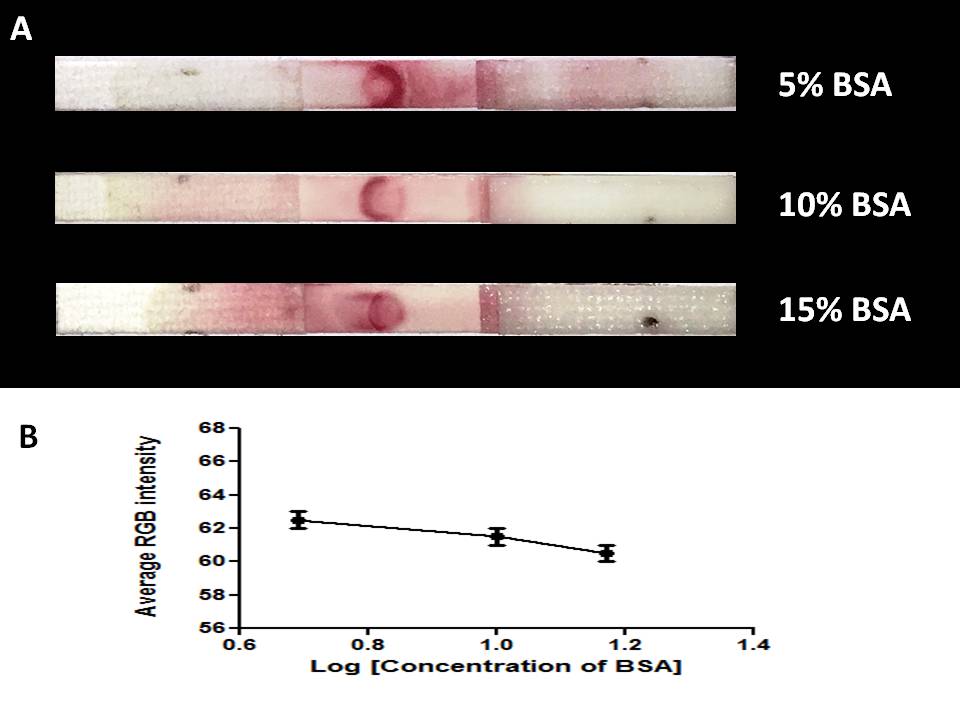


Fig S7. (A) Visual detection of BSA at its gradually increasing concentrations (5-15 mg/mL) in competitive format through NALFA where 10 µM aptamer was immobilized on NC laminate, and AuNPs- CA125 conjugate was applied on reservoir matrix. The assay was run using varying concentrations (5-15 mg/mL) of BSA in 0.1M phosphate buffer as the test sample (N=4). (B) Quantification of NALFA using ImageJ software by calculating average RGB intensities.

| **Name** | **Sequence** | **Frequency** |
| --- | --- | --- |
| Apt 2.26 | 5'TAGGGAAGAGAAGGACATATGATTTTAGGGAAGAGAAGGACTTTTATGCCGCCTTGACTAGTACATGACCACTTGA 3' | 27 |
| Apt 2.43 | 5'TAGGGAAGAGAAGGACATATGATGATCAACAACACAAGGGGGGGGGTATCTAGTTGACTAGTACATGACCACTTGA 3' | 13 |
| Apt 2.13 | 5'TAGGGAAGAGAAGGACATATGATGGGCGGACAGCTGGGCTAGTGATTGGTACGTTGACTAGTACATGACCACTTGA 3' | 9 |
| Apt 1.10 | 5'TAGGGAAGAGAAGGACATATGATGGTGCGATGTAGAGGCCACCCCTCGGCCATTTGACTAGTACATGACCACTTGA 3' | 2 |

Table S1. Sequence information of aptamer candidates in the order of their frequency
